# Hospital Audit as a Useful Tool in the Process of Introducing Falsified Medicines Directive (FMD) into Hospital Pharmacy Settings—A Pilot Study

**DOI:** 10.3390/pharmacy5040063

**Published:** 2017-11-09

**Authors:** Urszula Religioni, Damian Swieczkowski, Anna Gawrońska, Anna Kowalczuk, Mariola Drozd, Mikołaj Zerhau, Dariusz Smoliński, Stanisław Radomiński, Natalia Cwalina, David Brindley, Miłosz J. Jaguszewski, Piotr Merks

**Affiliations:** 1 Department of Public Health, Medical University of Warsaw, Banacha 1a, 02-097 Warsaw, Poland; urszula.religioni@gmail.com; 2 First Department of Cardiology, Medical University in Gdansk, Dębinki 7, 80-952 Gdańsk, Poland; ncwalina@gumed.edu.pl (N.C.), mjaguszewski@gumed.edu.pl (M.J.J.); 3 Institute of Logistics and Warehousing, Ewarysta Estkowskiego 6, 61-755 Poznań, Poland; anna.gawronska@ilim.poznan.pl; 4 National Medicines Institute, Chełmska 30/34, 00-725 Warsaw, Poland; a.kowalczuk@nil.gov.pl; 5 Department of Applied Pharmacy, Medical University of Lublin, Chodźki 1, 20-093 Lublin, Poland; marioladrozd@umlub.pl; 6 Hospital Pharmacy, Mazowiecki Szpitala Specjalistyczny w Ostrołęce, Al. Jana Pawła II 120 A, 07-410 Ostrołęka, Poland; mikizerh@gmail.com; 7 Community Pharmacy, Poland; smolinski.d.a@gmail.com; 8 Quizit Sp. z. o. o., Unit Dose, Sprinterów 2/6, 94-022 Łódź, Poland; s.radominski@quizit.eu; 9 Department of Paediatrics, University of Oxford, Level 2, Children’s Hospital, John Radcliffe, Headington, Oxford OX3 9DU, UK; david.brindley@paediatrics.ox.ac.u; 10 The Oxford—UCL Centre for the Advancement of Sustainable Medical Innovation (CASMI), The University of Oxford, Tavistock Square, London WC1H 9JP, UK; 11 Centre for Behavioural Medicine, UCL School of Pharmacy, University College London, BMA House, Tavistock Square, London WC1H 9JP, UK; 12 Harvard Stem Cell Institute, Cambridge, MA 02138, USA; 13 USCF-Stanford Centre of Excellence in Regulatory Science and Innovation (CERSI), San Francisco, CA 94158, USA; 14 Department of Pharmaceutical Technology, Faculty of Pharmacy Collegium Medicum in Bydgoszcz, Nicolaus Copernicus University in Torun, 85-089 Bydgoszcz, Poland; piotrmerks@gmail.com

**Keywords:** hospital pharmacy, pharmacist, pharmaceutical law, point of authentication, patient safety, Falsified Medicines Directive

## Abstract

Background: Recently, the European Union has introduced the Falsified Medicines Directive (FMD). Additionally, in early 2016, a Delegated Act (DA) related to the FMD was published. The main objective of this study was to evaluate the usefulness of external audits in the context of implementing new regulations provided by the FMD in the secondary care environment. Methods: The external, in-person workflow audits were performed by an authentication company in three Polish hospital pharmacies. Each audit consisted of a combination of supervision (non-participant observation), secondary data analysis, and expert interviews with the use of an independently designed authorial Diagnostic Questionnaire. The questionnaire included information about hospital drug distribution procedures, data concerning drug usage, IT systems, medication order systems, the processes of medication dispensing, and the preparation and administration of hazardous drugs. Data analysis included a thorough examination of hospital documentation in regard to drug management. All data were subjected to qualitative analysis, with the aim of generating meaningful information through inductive inference. Results: Only one dispensing location in the Polish hospitals studied has the potential to be a primary authentication area. In the audited hospitals, an Automated Drug Dispensing System and unit dose were not identified during the study. Hospital wards contained an enclosed place within the department dedicated to drug storage under the direct supervision of senior nursing staff. An electronic order system was not available. In the largest center, unused medications are re-dispensed to different hospital departments, or may be sold to various institutions. Additionally, in one hospital pharmacy, pharmacists prepared parenteral nutrition and chemotherapeutic drugs for patients admitted to the hospital. Conclusions: External audits might prove beneficial in the course of introducing new regulations into everyday settings. However, such action should be provided before the final implementation of authentication services. To sum up, FMD can impact several hospital departments.

## 1. Introduction

According to the World Health Organization (WHO), approximately 10% of medicinal products available within legal distribution are falsified. The financial value of this market reaches over 45 billion Euros, when considered in a global perspective. This problem remains underrecognized, particularly in highly-developed countries, where experts estimate that ca. 50% of drugs purchased via the Internet are falsified [1,2,3,4]. For the time being, the most frequently falsified medicines are those for weight loss and erectile dysfunction, as well as steroid hormones [5,6,7]. Recently, authorities have identified an increased number of falsified medicines in other therapeutic groups, such as contraceptives, psychotropic drugs, or cardiovascular agents [8,9,10].

The falsification of medicinal products remains an international issue, and thus it is necessary for the global authorities that are responsible for the creation of regulation frameworks, e.g., the Food and Drug Administration (FDA) or the European Medicines Agency (EMA), to implement actions against this. Recently, the European Union introduced the Falsified Medicines Directive (FMD) [11]. Additionally, in early 2016, a Delegated Act (DA) related to the FMD was published. The DA included additional details and administrative procedures related to the new regulations. This information is particularly important for stakeholders involved in the pharmaceutical supply chain [12,13,14]. The most fundamental changes are based on authentication, i.e., the scanning of a unique identifier during the moment of drug dispensation. This step in authentication is associated with a new range of responsibilities for pharmacy staff [12]. The detailed regulations include a list of the drugs which must have a unique identifier, as well as a list of those which do not require authentication [12]. However, the differences between verification and authentication should be emphasized. Broadly speaking, drug verification could be performed at any time during drug distribution. Authentication leads to the withdrawal of unique identifiers from the system and should be done directly before the final dispensation [12]. All stakeholders involved in drug distribution both at the national and European level should create a depository of unique identifiers. This will require mutual understanding and effective cooperation [12]. These actions should be understood as an important step in ensuring the safety of European patients. Based on this regulation, hospital pharmacies in Europe have until February 2019 to launch authentication protocols and to complete successful harmonization with the new legislature [15,16]. One of the essential steps for the effective implementation of these regulations into routine practice subsets remains the introduction and continuous improvement of validated procedures, using Good Authentication Practice (GAP) [17].

So far, the implementation of FMD has been discussed in the context of the community pharmacy setting. However, as stated by Merks et al., the introduction of new regulations into hospital pharmacies might prove to be even more challenging [18]. For instance, it is necessary to find the most suitable place for decommissioning (withdrawal of a unique identifier from the system) and all new procedures must be harmonized with the existing framework used [18]. In Poland, hospital pharmacies are mainly responsible for drug distribution and as such, clinical activities are rare [19]. Hospitals pharmacists develop the list of drugs used in a particular hospital (under the direct supervision of physicians), order drugs from wholesalers, and supervise drug management on hospital wards [19]. From this perspective, hospital pharmacies are the center of creation for drug policies in hospitals [20]. Moreover, hospital pharmacists often prepare compounding formulations, e.g., pediatric dosages, chemotherapeutic agents, and intravenous medications including parenteral nutrition [19,20]. The head of the hospital pharmacy must be a fully qualified pharmacist with a specialization in hospital pharmacy and/or proper experience, as specified by Polish pharmaceutical law [21]. Depending on the hospital pharmacy location (for instance a regional or highly specialized medical center), the pharmacist’s responsibilities might be different, e.g., not all hospitals prepare chemotherapeutic agents [18,19,20].

We evaluated the potential usefulness of external audits in regard to the implementation of the new regulations provided by the FMD into the routine hospital setting. Audits remain an important step in the process of understanding the impact of the FMD on the workflow within the hospital pharmacy setting.

## 2. Materials and Methods

In 2015, the research team conducted three independent audits in hospitals located in central Poland: two with <300 beds and one with >500 beds (convenience sampling). Each audit was followed by a second audit, which was performed by an external company experienced in authentication services. Each of the three hospitals has their own hospital pharmacy, which is not an obligatory prerequisite required by Polish pharmaceutical law. Audits focused on places in the hospital which may have a potential importance for the implementation of the FMD. In detail, a mixed-methods analysis, which included in-person workflow audits, was used. The collection of all relevant data was linked with chain distribution in the audited institutions. Each audit consisted of a combination of supervision (non-participant observation), secondary data analysis, and expert interviews with the use of an independently designed authorial Diagnostic Questionnaire. Data analysis included a thorough examination of hospital documentation in regard to drug management. This comprised of: hospital drug distribution procedures, data concerning drug usage, IT systems, medication order systems, the processes of medication dispensing, and the preparation and administration of hazardous drugs. The Diagnostic Questionnaire provided a general overview of the characteristics of each hospital pharmacy. All data was subjected to qualitative analysis, with the aim of generating meaningful information through inductive inference. This comprehensive analysis aided in obtaining specific and intersubjective features of the entire process. The supervision, secondary data analyses, and interviews allowed us to prepare reports that present optimal solutions for authentication in pharmacies. Finally, we provided recommendations for further steps in introducing FMD into the routine setting within the Polish healthcare system.

Ethics approval and consent to participate were not applicable.

## 3. Results

In the audited hospitals, no Automated Drug Dispensing Systems were identified within the pharmacy setting. It was documented that pharmacists did not dispense medications using a unit dose system. The hospital's drug supply was based on a standard chain distribution, with the majority of drugs purchased directly from their official producer or general wholesalers. The area of dispensing medicinal products in the hospitals was considered as a potential place of authentication. However, in the hospital with >500 beds, we identified possible additional methods of integration in using an authentication system, such as the use of mobile scanners. Hospital pharmacists often utilized shared packages, namely split packs, and for the most part, drugs were dispensed in bulk. Two of the audited hospital pharmacies did not provide compounded intravenous medications. One of the settings, however, did provide parenteral nutrition and chemotherapeutic preparations. Hospital wards were not directly supplied by the producers or wholesalers; the drug chain distribution was based on medicinal products available within the hospital pharmacies. On the hospital wards, we identified enclosed places that were dedicated to drug storage, and maintained under the direct supervision of senior nursing staff. An electronic ordering system was not used by any of the hospital wards, departments, or clinics. Ordering was based on traditional paper communication between wards and hospital pharmacies as authorized by the head of departments. In line with their internal protocols, hospital wards returned unused medications to the hospital pharmacy. The returned drugs could then be dispensed to other departments in the hospital, depending on current needs. However, drug suppliers required that medications were returned to the hospital pharmacy within seven days of dispensing, and that this prerequisite was fulfilled in an audited setting. All audited hospitals were part of a hospital network and coordinated broad-spectrum services within the group of institutions. The transition of medicinal products between various hospitals did not occur in any of the audited bodies. The hospitals did not use an external, outsourced administration. The results of our audits are summarized in Table 1.

## 4. Discussion

To the best of our knowledge, this is the first report attempting to evaluate the usefulness of hospital audits during the process of FMD implementation within the hospital pharmacy, especially in countries where the pharmacist’s position is limited to dispensing medications and where there is no history of well-established pharmaceutical care. We found that dispensaries are the most optimal places of authentication; however, we suggest that additional automated technology may be beneficial in larger hospital settings, e.g., those with more than 500 beds. We recommend that any unused medications be returned from the hospital wards to the pharmacy within 10 days from the moment of dispensation. Internal operation procedures need to be implemented. In this way, pharmacy staff can re-introduce the serialized unique identifier through the authentication system and decommission the unique identifier again when the package is dispensed. If more than 10 days have passed since the authentication was performed, the drug can still be returned to the hospital pharmacy, but can only be used in the physical institution that conducted the decommission operation. It should not be sold to other institutions or returned to the supplier. In our work, the audited hospitals did not use a more advanced distribution system e.g., unit dose. We should stress that this tendency is in accordance with previous studies in this field and noted as a national trend [22].

Moreover, we have provided some general recommendations on how to harmonize authentication with the standards currently used in hospital settings. All hospitals were considered as large. Due to this distinction, it is important to optimize internal procedures to ensure that products are not authenticated too early in the supply chain. Once medications are authenticated and a period of 10 days has elapsed, these products can no longer be returned to a manufacturer nor can they be transferred to another institution. In the context of dispensing drugs in bulk, we recommend selecting products from manufacturers that can produce aggregated codes, as this will reduce the time needed to authenticate products. Due to the fact that hospital pharmacies often use split packs, authentication must be conducted prior to opening a drug package, and the total content of the product needs to be kept at all times within the controlled environment of the hospital pharmacy. Of course, in this scenario, drugs cannot be transferred to any other area of the hospital, or be re-sold or transferred to another institution.

Ward stock can be maintained, as there is no need to change internal procedures within the hospital. If the hospital belongs to a trust/group of hospitals, and they legally all belong to the same institution, the authentication process can be performed in one of the hospital pharmacies. Drugs returned from hospital wards can be disposed of, if this is the normal policy of the hospital, or they can be reused, but only inside the same physical institution if the 10-day rule has passed. They cannot be re-sold or returned to the manufacturer.

Authentication of extemporaneously prepared medicines, intravenous and parenteral nutrition products, etc., should occur while assembling ingredients for the product before the final product is prepared by a qualified member of staff. A proper system to dispose of medicinal products needs to be put in place. The risk of reusing the packaging of products can be substantial depending on the setting. To summarize, authentication needs to be performed within the hospital pharmacy. Detailed records must be kept due to the possibility of product recall. Above mentioned recommendations are summarized in Table 2. 

We believe that the implementation of FMD should be associated with amendments in the management of hospital pharmacies, as it will place new responsibilities on pharmacists and technicians employed within hospital pharmacies. Standard procedures should be introduced and harmonized with European laws, and further adjusted to the national practice [23]. Additionally, there is a strong need to adapt the protocols to the workflow available in a particular community or hospital pharmacy [24].

Here, we would like to stress the fact that GAP can be helpful in the successful implementation of a new framework for hospital pharmacies [17,25]. In line with GAP, our study suggested that the most desirable authentication should be provided by hospital pharmacists or technicians, at the final step of dispensing medications. Decommissioning procedures also need to occur. Moreover, whenever possible, manual authentication should be replaced by an automated process. By using scanners that read a 2D matrix, the risk of releasing a drug without authentication can be reduced, in accordance with lean management concepts. While legal regulations allow for the division of a drug package, the original container should not leave the dispensing point until its entire contents have been dispensed. Also, in instances when the hospital pharmacy contains a chemotherapeutic facility for compounding, the authentication of such products must be obtained before the drug is introduced in the clean room [17].

Our observations have several potential limitations. First, the process of drug authentication in the hospital setting remains in the early stages of development. A standard of best practices can only be achieved over time with the acquisition of experience and further conducted research. Secondly, our initial audits have been conducted only in three hospital pharmacies. Consequently, our findings cannot serve as an entirely accurate representation of all hospital pharmacies in Poland. However, we would like to emphasize that this is only a pilot study and again, further investigation is warranted. Moreover, we are still waiting for recommendations approved by national authorities, which will specify procedures in light of countrywide requirements [26].

## 5. Conclusions

FMD should be fully harmonized with national legal framework in accordance with the 2019 implementation deadline [18]. Currently, hospital pharmacies in Poland are not organized under the new regulations associated with the FMD [27]. The joint efforts of both pharmacists and hospital management staff are essential to the effective implementation of an innovative workflow in the hospital pharmacy setting. A pilot program of authentication should precede the final implementation. Preparations of standard operational procedures that are adjusted to national practice might prove very useful. External audits can be beneficial in this process and should be performed before introducing new tools into routine settings.

## Figures and Tables

**Table 1:** A summary of the results of audits.

Hospital	The Number of Hospitals Beds	Audits—Results
1 and 2	<300	Both hospitals had their own hospital pharmacy. We did not identify an Automated Drug Dispensing System nor the use of a unit dose. Hospital wards contained an enclosed place within the department dedicated to drug storage under the direct supervision of senior nursing staff. An electronic order system was not available. Hospital pharmacies did not provide compounded intravenous medications. Unused medications might be re-dispensed to different hospital departments. The hospitals are part of a hospital network; however, the individual hospital pharmacies did not provide drugs to different institutions.
3	>500	In this hospital there was only one hospital pharmacy. We did not identify an Automated Drug Dispensing System nor a unit dose distribution model. On the hospital wards, there was an enclosed place dedicated to drug storage under the direct supervision of senior nursing staff. An electronic order system was not available.

**Table 2:** A summary of recommendations.

	Recommendations
1	Dispensaries are the most optimal places of authentication; however, additional automated technology may be beneficial in larger hospital settings, e.g., those with more than 500 beds.
2	Any unused drugs are to be returned from the hospital wards to the pharmacy within 10 days from the moment of dispensation.
3	If more than 10 days have passed since the authentication has been performed, the drug can still be returned to the hospital pharmacy, but can only be used in the physical institution that conducted the decommission operation.
4	Authentication needs to be conducted before opening a drug package, and the total content of the product needs to be kept at all times within the controlled environment of the hospital pharmacy.
5	If the hospital belongs to a trust/group of hospitals, the authentication process can be done in one of the hospital pharmacies.
6	Authentication of extemporaneously prepared medicines, intravenous, and parenteral nutrition products, etc., should occur while assembling ingredients for the product before the final product is prepared by a qualified member of staff.
7	Authentication needs to be performed in the hospital pharmacy.
8	It is important to optimize internal procedures to ensure that products are not authenticated too early in the supply chain.
